# YY1 targets tubulin polymerisation-promoting protein to inhibit migration, invasion and angiogenesis in pancreatic cancer via p38/MAPK and PI3K/AKT pathways

**DOI:** 10.1038/s41416-019-0604-5

**Published:** 2019-10-21

**Authors:** Qun Chen, Chuang Yang, Lei Chen, Jing-Jing Zhang, Wan-Li Ge, Hao Yuan, Ling-Dong Meng, Xu-Min Huang, Peng Shen, Yi Miao, Kui-Rong Jiang

**Affiliations:** 1 Pancreas Center, the First Affiliated Hospital of Nanjing Medical University, Nanjing, China; 2 Pancreas Institute, Nanjing Medical University, Nanjing, China

**Keywords:** Cancer epigenetics, Metastasis

## Abstract

**Background:**

Pancreatic cancer (PDAC) is a highly invasive cancer with poor prognosis. Recent research has found that the transcription factor Yin Yang 1 (YY1) plays an inhibitory role in the development of pancreatic cancer. It has been reported that tubulin polymerisation-promoting protein (TPPP) plays an indispensable role in a variety of tumours, but its expression and role in pancreatic cancer have not yet been elucidated.

**Methods:**

In this study, we performed ChIP-sequencing and found that YY1 directly binds to the promoter region of TPPP. The expression of TPPP in pancreatic cancer was detected by western blotting and immunohistochemistry. Four-week-old male BALB/c-nude mice were used to assess the effect of TPPP on pancreatic cancer.

**Results:**

Immunohistochemistry revealed that TPPP was expressed at low levels in pancreatic cancer tissues, and was associated with blood vessel invasion. The results from vivo experiments have showed that TPPP could enhance the migration and invasion of pancreatic cancer. Further experiments showed that YY1 could inhibit the migration, invasion and angiogenesis of pancreatic cancer cells by downregulating TPPP via p38/MAPK and PI3K/AKT pathways.

**Conclusion:**

Our study demonstrates that TPPP may act as a promoter and may serve as a novel target for the treatment of pancreatic cancer.

## Background

Pancreatic cancer (PDAC), the fourth leading cause of cancer-related deaths worldwide, is difficult to diagnose early and is highly malignant.^1,2^ The 5-year survival rate is <8%, while that in patients with PDAC after surgery is <26%.^3,4^ PDAC is not sensitive to radiotherapy or chemotherapy, and easily invades and metastasises to lymph nodes or adjacent organs.^5^ Therefore, there is an urgent need to study the cellular and molecular characteristics of PDAC and to explore new diagnostic and therapeutic targets.

The transcription factor Yin Yang 1 (YY1) is a member of the GLI-Kruppel family of nuclear proteins that plays an important role in various biological activities, including cell proliferation, angiogenesis and metastasis.^6–9^ Our previous studies have shown that YY1 is highly expressed in pancreatic cancer, but exerts an inhibitory effect.^10^ According to the previous ChIP-sequencing results, YY1 binds to the promoter region of tubulin polymerisation-promoting protein (TPPP) and regulates its expression, indicating that TPPP may be involved in the development of pancreatic cancer.^11^

TPPP is a member of the tubulin polymerisation-promoting protein family, a novel MAP family, that is expressed primarily in the brain and neuroblastoma cells.^12–14^ TPPP promotes tubulin assembly and blocks the formation of mitotic spindles.^15^ In recent years, research on TPPP in tumours has increased. TPPP was found to be highly expressed in colorectal cancer, lung cancer and bladder cancer, but was found to be expressed at low levels in liver cancer.^16–19^ To the best of our knowledge, TPPP has not been reported in pancreatic cancer, so it is necessary to further study its important role in pancreatic cancer.

In our research, we delved into the role of TPPP induced by YY1 in the development and progression of pancreatic cancer. TPPP promotes the migration, invasion and angiogenesis of pancreatic cancer through the p38/MAPK and PI3K/AKT signalling pathways.

## Methods

### Tissue microarrays and immunohistochemistry

This study was approved by the Ethics Committee of the First Affiliated Hospital of Nanjing Medical University, and all patients provided written informed consent. To verify the expression of TPPP in PDAC tissues, 71 pancreatic cancer tissues and their corresponding tissue microarrays (TMAs) were obtained from Shanghai Zhuoli Biotechnology Co., Ltd. (Zhuoli Biotechnology Co., Shanghai, China). Tumour differentiation was assigned according to the standards of the World Health Organization standards. All tissue samples were fixed in formalin and embedded in paraffin for future use in IHC.

Immunohistochemistry was performed, as we previously reported.^20^ The sections were incubated overnight at 4 °C with an anti-TPPP polyclonal antibody (#ab92305, Abcam, Cambridge, MA, 1:250 dilution), followed by an incubation with a secondary antibody (goat anti-rabbit/horseradish peroxidase, Santa Cruz Biotechnology, 1:200 dilution) at room temperature for 30 min. Finally, the sections were visualised with diaminobenzidine for ~5 min and counterstained with haematoxylin. Two independent pathologists evaluated the immunohistochemical data for the TPPP levels. The expression levels were assessed based on the staining intensity (0 for no staining, 1 for weak, 2 for moderate, 3 for strong staining) and the positive cell ratio (0 for <10%, 1 for 10 to <50%, 2 for ≥50% of cells). The two scores were combined with the following formula: IHC score = positive rate score × intensity score.

### Cell lines and culture

Four human pancreatic cancer cell lines (MiaPaCa-2, BxPC-3, CFPAC-1 and PANC-1) and a normal human pancreatic ductal cell line (HPNE) were purchased from the Shanghai Cell Bank (Shanghai, China). Cells stably overexpressing YY1 or with YY1 knockdown had already been transfected into the BxPC-3 and PANC-1 cell lines (BxPC-3-YY1, BxPC-3-YY1 shRNA, PANC-1-YY1, PANC-1-YY1 shRNA). The corresponding control cell lines (BxPC-3-Vector, BxPC-3-Scramble shRNA, PANC-1-Vector, PANC-1-Scramble shRNA) had also already been prepared.^21^

As described previously, PDAC cells were grown in Dulbecco’s modified Eagle’s medium (DMEM) (Life Technologies) supplemented with 10% foetal calf serum (FBS) (Wisent Inc., Montreal, Qc, Canada), 10 mM HEPES (Sigma, St. Louis, MO, USA), 2 mM L-glutamine (Sigma), 1 mM pyruvate sodium (Sigma), 100 U/ml penicillin (Life Technologies), and 100 μg/ml streptomycin (Life Technologies). HPNE cells were grown in keratinocyte serum-free medium supplemented with epidermal growth factor and bovine pituitary extract at 37 °C in a humidified atmosphere containing 95% air and 5% CO_2_^10^

### Preparation of TPPP-overexpressing cells

TPPP-overexpressing lentiviruses were constructed by Hanbio Biotechnology Co., Ltd. (Shanghai, China). The full-length coding region of human TPPP was sub-cloned into the HBLV-h-TPPP-3 × FLAG-GFP-PURO plasmid (System Biosciences, Mountain View, CA, USA). The verified recombinant vector and the pPACKH1 packaging plasmid (System Biosciences) were co-transfected into 293T cells using Lipofectamine 3000 reagent (Life Technologies). The supernatant of the cultured 293T cells was collected to infect BxPC-3 cells, BxPC-3-YY1 cells, PANC-1 cells and PANC-1-YY1 cells. The pHBLV-CMV-MCS-3 × FLAG-EF1-ZsGreen-T2A-PURO vector was used to package the virus and infect BxPC-3 cells, BxPC-3-YY1 cells, PANC-1 cells and PANC-1-YY1 cells as a control. Stable cell lines were selected by culturing in medium containing 5 μg/ml puromycin (Sigma). TPPP expression was confirmed by qRT-PCR and western blot.

### RNA isolation and quantitative real-time PCR

Briefly, the total RNA was isolated from cells using TRIzol Reagent (Life Technologies, Carlsbad, CA, USA). The total RNA was then reverse-transcribed with an iScript cDNA Synthesis Kit (Bio-Rad, Hercules, CA, USA). Complementary DNA synthesis and the quantitative real-time PCR were performed as our previously reported.^10^ Each quantitative PCR was performed in triplicate, and independently repeated three times.

### Western blotting

Protein was extracted from the cells and quantified by a Total Protein Extraction Kit (Keygen BioTech, Nanjing, China). Western blotting was performed according to the manufacturer’s protocol. Anti-TPPP, anti-YY1 (#ab109228), anti-E-cadherin (#ab40772), anti-vimentin (#ab92547), anti-MMP3 (#ab52915), anti-MMP7 (#ab205525), anti-VEGF (#ab32152), anti-p38 (#ab170099), anti-MAPK (#ab205926), anti-p38 MAPK (phosphor, Thr180/Tyr182, #4511S), anti-PI3K (#4255S), anti-PI3K (phosphor, Ser249, #13857S), anti-AKT (#2920S), anti-AKT (phosphor, Thr308, #13038S), anti-β-actin (#3700S) and anti-YY1 (#ab12132) antibodies for ChIP were obtained from Abcam (Cambridge, MA) or Cell Signaling Technology (Danvers, MA, USA). β-Actin was used as an endogenous reference. Each blot was independently repeated three times.

### Cell wound-healing assay for cell migration

Cell wound-healing assays were performed as our previously reported.^20^

### Cellular Transwell assay to test cell migration and invasion

As described previously, the cells were inoculated into serum-free medium in inserts with an 8-μm pore size (Corning) to assess migration and invasion.^20^ Each assay was repeated three times, and the mean number of cells in ten random fields per well (×100  magnification) were compared between groups. For Matrigel invasion assays, inserts with an 8 µm pore size were coated with 1 mg/mL growth factor-reduced Matrigel (BD Bioscience Pharmingen).

### Cell count kit-8 (CCK-8) and clone-formation assay to test cell proliferation

A total of 3 × 10^3^ control or transfected cells were added to each well in a 96-well plate to assess the effect of TPPP on cell proliferation. At the same time each day, 100 μl of medium containing 10 μl of CCK-8 (Dojindo, Japan) was added, and after incubating at 37 °C in the dark for 2 h, each well was analysed by a microplate reader with a wavelength of 450 nm. Each sample had five duplicate wells, and each experiment was independently repeated three times.

Cells were cultured for 14 days in DMEM supplemented with 10% FBS at 37 °C in a humidified atmosphere containing 95% air and 5% CO_2_. The medium was refreshed every 3 days. The colonies were stained with 0.1% crystal violet, and then the number of positively stained colonies was counted.

### HUVEC tube-formation assay to test cell angiogenesis

HUVECs (4 × 10^4^ cells/well) were seeded in 96-well plates coated with 200 μl Matrigel. The 24-well plates were incubated for 6 h, and tube formation was then photographed in five random fields under an inverted microscope. The endothelial tubes were quantified by counting the length and branches per image field using ImageJ software.

### Construction of reporter gene plasmids

A luciferase reporter construct containing the human TPPP promoter was prepared using the pGL3-Basic vector (Promega, Madison, WI, USA). A DNA fragment of the TPPP promoter region (including restriction enzyme sites) synthesised by GenScript Biotechnology Co., Ltd. (Nanjing, China) was sub-cloned into the KpnI and XhoI sites of the pGL3-Basic vector to construct the recombinant pGL3-TPPP-promoter (pTPPP) plasmid, which was confirmed by sequencing. A mutant construct pTPPP-YY1-M containing the TPPP promoter was also constructed, in which the putative mutation site is CGGGATGGTGGC.

### Cell transient transfection and luciferase assay

According to the manufacturer’s protocol, transfections were performed by Lipofectamine 3000. A total of 3 × 10^5^ cells were seeded into each well of six-well cell culture plates 1 day before transfection, as described above. Cells were transfected with 1 µg of the luciferase reporter plus 1 ng of the Renilla luciferase reporter vector pRL-SV40, which was used as an internal control each time. The cells were washed with PBS and lysed using 1 × passive lysis buffer after 48 h. Firefly and Renilla luciferase activities were measured with a Promega Dual-Luciferases Reporter Assay Kit (Promega) according to the protocol. Each experiment was performed in triplicate, and independently repeated three times.

### Chromatin immunoprecipitation (ChIP) assay

Chromatin immunoprecipitation (ChIP) was carried out with the Magna Chromatin Immunoprecipitation kit (Millipore, Darmstadt, Germany). Immunoprecipitation was performed with anti-YY1 antibody. The final purified DNA fragment was subjected to PCR analysis using Hot-Start Taq DNA polymerase (Takara, Dalian, China; 32 cycles). The primers used were as follows: sense ATTGCCACACAGGCGGACAG, antisense TGGATGAATGCTGGGAAGC. PCR products were analysed using gel electrophoresis. ChIP data are shown as the percentage of the input normalised to control purifications.

### Animal study

Four-week-old male nude mice (BALB/c-nu) were purchased from the Animal Center of Nanjing Medical University. All animal experiments were conducted in compliance with animal protocols approved by Nanjing Medical University, and were carried out at the Animal Center of Nanjing Medical University. Sixteen mice were randomly divided into two groups for the construction of a tail vein metastasis model. Stable cells (PANC-1-TPPP and PANC-1-TPPP vector) were prepared as described previously. Cells (1.5 × 10^6^ cells/100 μl) were injected into the tail vein of each mouse. The vital signs of mice were observed each week after injection, and no death was observed in the two groups. Four weeks later, the mice were euthanised with carbon dioxide, and the lungs and livers were removed, fixed with 4% paraformaldehyde, embedded in paraffin and sectioned. Sections were stained with H&E to assess the extent of metastasis in the lung and liver of tumour samples.

### Statistical analysis

All data are expressed as the mean ± standard deviation (SD) of three independent experiments. Comparisons between two groups were performed using Student’s *t* test. A Pearson chi-square test was performed to assess the relationship between TPPP expression and the clinicopathological features. The statistical analysis software package Stata (10.0) was used for statistical analysis, and *p* < 0.05 indicated statistical significance.

## Results

### Expression of TPPP in PDACs

**Fig. 1:**
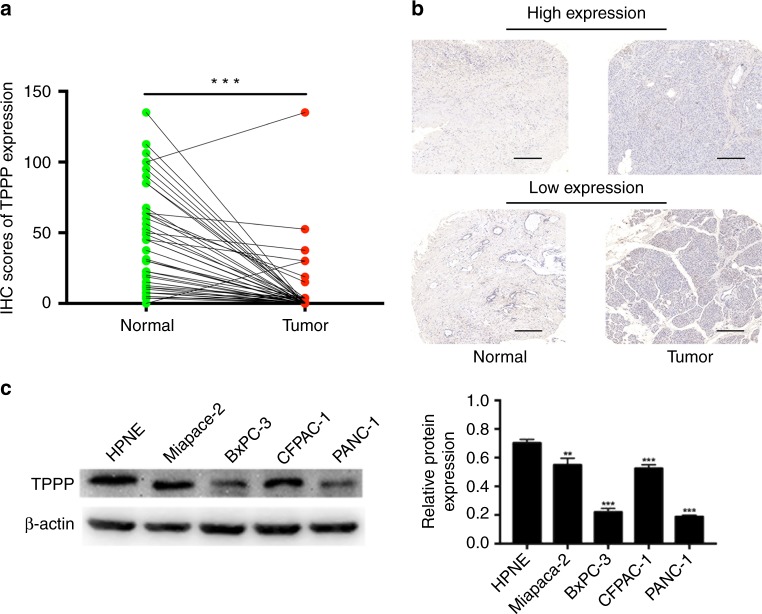
Expression of TPPP in PDAC. **a** A boxplot showing TPPP protein expression in 71 pairs of PDAC tissues and adjacent tissues, as determined by immunohistochemistry (IHC). **b** Less TPPP was expressed in PDAC cells than in adjacent tissues, as determined by IHC. Scale bar = 200 μm. **c** Western blot analysis showed TPPP protein expression in a panel of human PDAC cell lines and HPNE cells. (*** represents *p* < 0.001, compared with the control group)

In accordance with previous reports in the literature by Badea et al. and Ishikawa et al., we found that TPPP expression was decreased in pancreatic cancer tissues.^22,23^ Next, we performed IHC to detect the expression of TPPP in 71 pancreatic cancer tissues and adjacent tissues. We performed immunohistochemistry to evaluate the expression of TPPP between pancreatic cancer tissues and their adjacent tissues. The results shown that protein levels of TPPP were significantly lower in cancer tissues compared with that in adjacent tissues, which is consistent with the results of Badea et al.^22^ and Ishikawa et al.^23^ (Fig. 1a, b). We also analysed the association between the expression of TPPP and the clinicopathological features of these patients and found that expression of TPPP was associated with blood vessel invasion (*p* < 0.05) (Supplementary Tables S2, S3). All the results can be seen in Table 1. The expression of TPPP was also measured by a WB in four PDAC cell lines (MiaPaCa-2, BxPC-3, CFPAC-1 and PANC-1) and a normal human pancreatic ductal cell line (HPNE). Compared with HPNE cells, PDAC cells had a decreased expression level of TPPP (Fig. 1c). The PANC-1 and BxPC-3 cell lines were selected for subsequent studies.

**Table 1:** Association of TPPP expression with clinicopathological features of PDAC

Variable	Group	TPPP expression		*P-*value
		High	Low	
*Gender*				
	Male	5	40	0.081
	Female	8	18	
*Age (year)*				
	≤65	9	47	0.571
	>65	4	11	
*TNM stage*				
	I-IIA	10	37	0.562
	IIB-IV	3	21	
*Diameter (cm)*				
	≤3	4	23	0.779
	>3	9	35	
*Location*				
	Head	10	40	0.817
	Body, tail	3	18	
*T stage*				
	T1 or T2	12	51	1.000
	T3 or T4	1	7	
*N stage*				
	Absent	10	39	0.726
	Present	3	19	
*M stage*				
	Absent	11	53	0.822
	Present	2	5	
*Blood vessel invasion*				
	Absent	11	30	0.030*
	Present	2	28	
*Histological grade*				
	I/I–II/II	9	37	0.960
	II–III/III	4	21	

*TNM* stage: tumour–node–metastasis

*p < 0.05

### TPPP promotes the migration, invasion and angiogenesis of pancreatic cancer cells in vitro

**Fig. 2:**
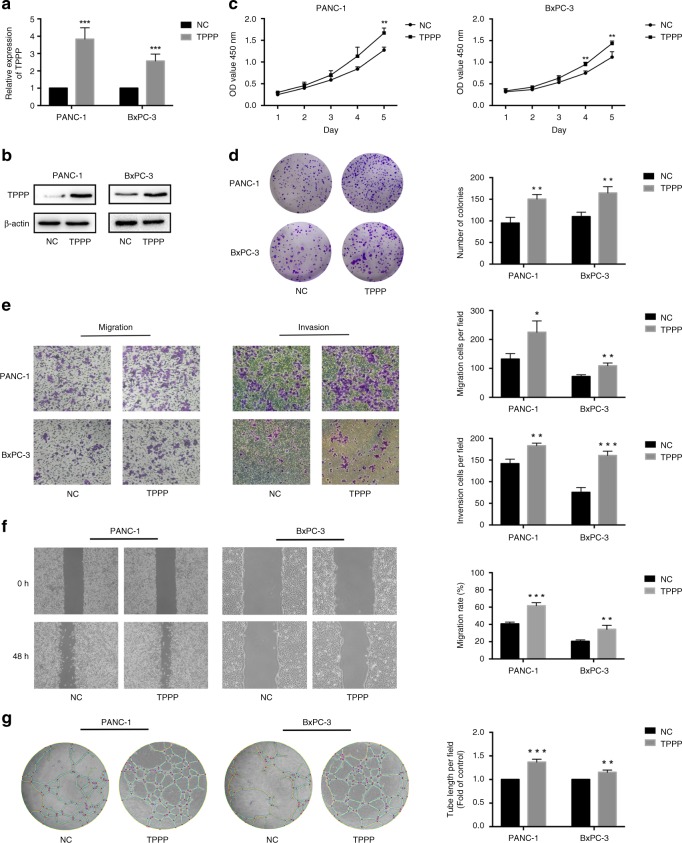
TPPP promotes the proliferation, migration, invasion and angiogenesis of pancreatic cancer cells. **a**, **b** The TPPP expression in TPPP-overexpressing PANC-1 cells and BxPC-3 cells was measured by quantitative RT-PCR and western blot. **c**, **d** CCK-8 assays and wound-formation assays were performed to analyse proliferation in the PANC-1-TPPP and BxPC-3-TPPP cells and in their corresponding control cells. **e** Cell migration and invasion assays were performed. PANC-1 and BxPC-3 cells transfected with TPPP-overexpressing lentiviruses and control lentiviruses. The membranes in the chambers were stained with 0.1% crystal violet. Scale bar, 100 μm. **f** Wound-healing assays were performed. PANC-1 and BxPC-3 cells were transfected with TPPP-overexpressing lentiviruses and control lentiviruses for 0 and 48 h. Magnification, ×200; scale bar, 100 μm. **g** HUVEC tube-formation assays were performed. Representative images of capillary-like structures stimulated by conditioned medium are shown. (*represents *p* < 0.05, **represents *p* < 0.01, *** represents *p* < 0.001, compared with the control group)

To investigate the effects of TPPP on pancreatic cancer cell function, we used viruses to transfect BxPC-3 and PANC-1 cells to obtain stable cell lines overexpressing TPPP. As shown, qRT-PCR and WB confirmed the expression levels of TPPP (Fig. 2a, b).

The effect of TPPP on pancreatic cancer cell proliferation was investigated by CCK-8 and clone-formation assays. Compared with that of the control cells, the absorption of the PANC-1-TPPP and BxPC-3-TPPP cells at OD450 was significantly increased (Fig. 2c). In addition, similar results were obtained in the cell clone-formation assay; that is, the number of colonies overexpressing TPPP was significantly higher in PANC-1-TPPP and BxPC-3-TPPP cells than in the control cells (Fig. 2d). These results indicate that the overexpression of TPPP can promote the proliferation of pancreatic cancer cells.

Cellular Transwell assays and wound-healing assays were used to assess the effects of TPPP on the invasion and migration of pancreatic cancer cells. The results of the Transwell assays showed that TPPP overexpression promoted the migration and invasion of pancreatic cancer cells (Fig. 2e). The results of the wound-healing assays were the same; TPPP overexpression accelerated the rate of wound healing (Fig. 2f). These results indicate that TPPP overexpression can promote the migration and invasion of pancreatic cancer cells.

We also assessed the effect of TPPP on the angiogenesis of pancreatic cancer cells. The conditioned medium with TPPP-overexpressing cells resulted in a significant increase in the number of tubular structures formed in HUVECs compared with those formed in the control cells (Fig. 2g). These results showed that TPPP overexpression can promote angiogenesis in pancreatic cancer cells.

### Interaction between TPPP and YY1

**Fig. 3:**
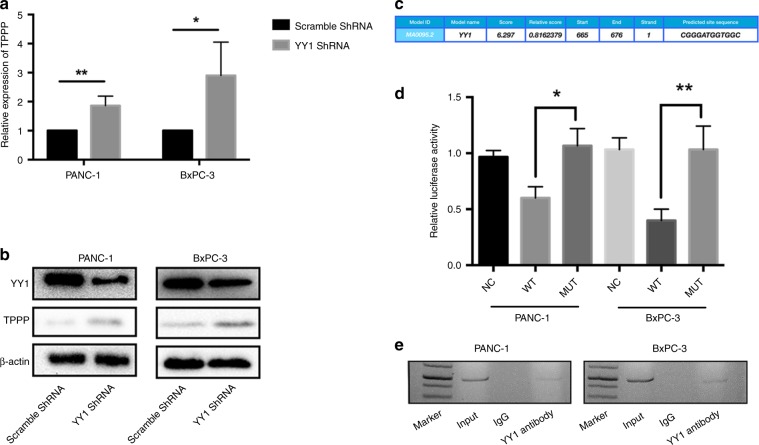
YY1 combined with the promoter region of TPPP regulates the expression of TPPP. **a**, **b** The TPPP expression in PANC-1-YY1 shRNA and BxPC-3-YY1 shRNA cells was measured by quantitative RT-PCR and western blot. **c** The cchematic diagram of the luciferase reporter construct containing the human TPPP promoter (pTPPP) and the mutant construct (pTPPP-YY1-M) containing the TPPP promoter in which the presumed YY1 binding site was mutated. **d** Luciferase assays demonstrating the luciferase activity of pTPPP (NC, WT or MUT) in PANC-1 (left) and BxPC-3 (right) cells transfected with YY1-overexpressing lentiviruses or control lentiviruses. Each error bar indicates the variation between the means of three independent experiments. **e** ChIP assays were performed in PANC-1-YY1 (left) and BxPC-3-YY1 (right) cells. Lane 1, DNA marker; lane 2, input DNA; lane 3, DNA from BxPC-3-YY1 cells immunoprecipitated with normal rabbit IgG; lane 4, DNA from BxPC-3-YY1 cells immunoprecipitated with an anti-YY1 antibody. (*represents *p* < 0.05, **represents *p* < 0.01, compared with the control group)

According to the previous ChIP-seq data, the YY1 transcription factor may bind to the promoter region of TPPP and regulate the transcription of TPPP.^11^ According to the results of qRT-PCR and WB, we found that the expression of TPPP was upregulated in BxPC-3-YY1 shRNA and PANC-1-YY1 shRNA cells compared with its expression in the control cells (Fig. 3a, b). To elucidate whether the promoter region of TPPP binds to YY1, we designed a TPPP reporter gene plasmid for luciferase assays and ChIP assays for in vivo validation.

For the luciferase experiments, YY1-overexpressing plasmids and reporter plasmids were co-transfected into PANC-1-YY1 and BxPC-3-YY1 cells. The overexpression of YY1 significantly reduced luciferase activity compared with that observed in the control group. Meanwhile, when the putative binding site of YY1 (nucleotides 665–676) was mutated, the luciferase activity of the TPPP promoter in YY1-overexpressing cells was increased compared with that observed in the control cells (Fig. 3d). These results indicate that there is a specific binding site for YY1 in the TPPP promoter region.

For the ChIP assay, DNA in the PANC-1-YY1 and BxPC-3-YY1 cells was extracted and purified. Specific primers containing the YY1-binding site were designed for qRT-PCR. As shown in Fig. 3e, PCR products were found in the YY1 immunoprecipitated group in both PANC-1 and BxPC-3 cells. These results indicate that the TPPP promoter and YY1 interact in vivo.

### TPPP is regulated by YY1

**Fig. 4:**
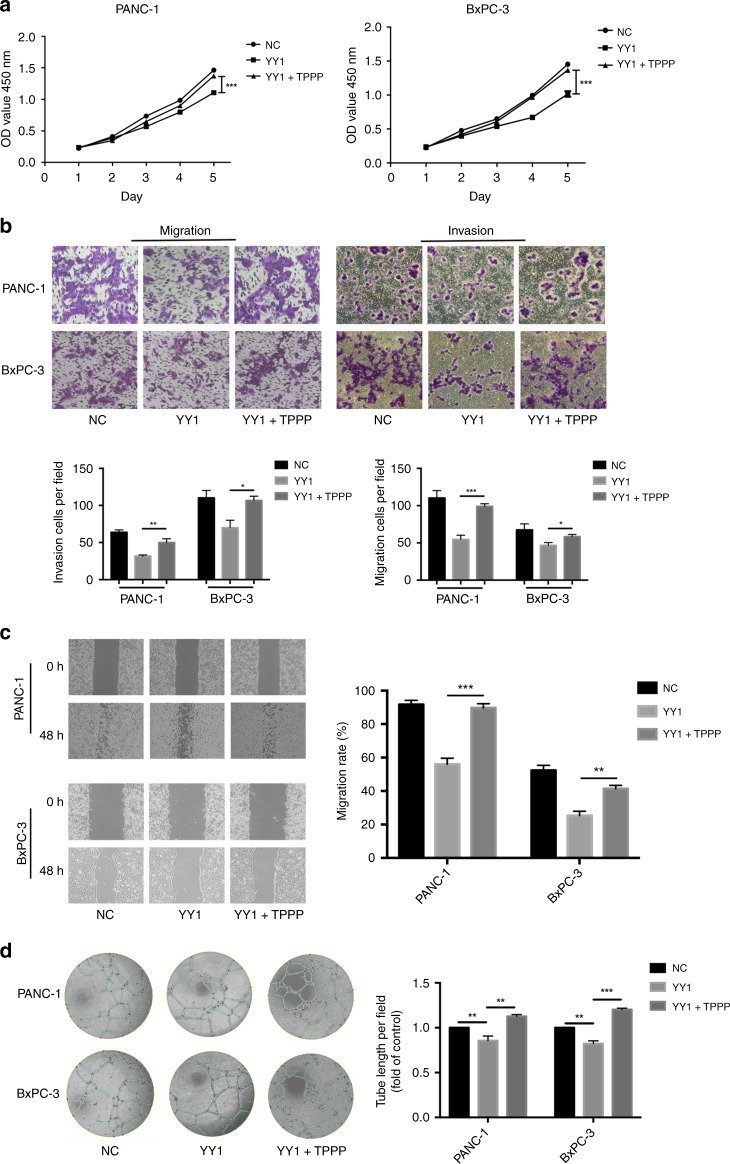
TPPP is a functional target of YY1. **a** CCK-8 assays were performed to analyse proliferation in PANC-1-YY1 (left) or BxPC-3-YY1 (right) cells transfected with TPPP-overexpressing lentiviruses or control lentiviruses. **b** Cell migration and invasion assays were performed. PANC-1-YY1 or BxPC-3-YY1 cells transfected with TPPP-overexpressing lentiviruses or control lentiviruses. The membranes in the chambers were stained with 0.1% crystal violet. Scale bar, 100 μm. **c** Wound-healing assays were performed. PANC-1-YY1 or BxPC-3-YY1 cells were transfected with TPPP-overexpressing lentiviruses or with control lentiviruses for 0 and 48 h. Magnification, ×200; scale bar, 100 μm. **d** HUVEC tubeformation assays were performed. Representative images of capillary-like structures stimulated by conditioned medium are shown. (*represents *p* < 0.05, **represents *p* < 0.01, ***represents *p* < 0.001, compared with the control group)

Previous experiments have found that the overexpression of YY1 inhibits the proliferation, migration and metastasis of pancreatic cancer cells.^24^ We obtained consistent results and found that the overexpression of YY1 inhibited angiogenesis in BxPC-3 and PANC-1 cells (Fig. 4d).

The results of CCK-8 assays showed that the upregulation of TPPP in YY1-overexpressing cells restored the inhibition of BxPC-3 and PANC-1 cell proliferation by YY1 (Fig. 4a). Cellular Transwell assays and wound-healing assays showed that the upregulation of TPPP restored the inhibitory effect of YY1 overexpression on the invasion and migration of pancreatic cancer cells (Fig. 4b, c). Finally, we found that the upregulation of TPPP also restored the inhibitory effect of YY1 overexpression on pancreatic cancer angiogenesis (Fig. 4d).

### TPPP affects the migration and invasion of pancreatic cancer cells in vivo

**Fig. 5:**
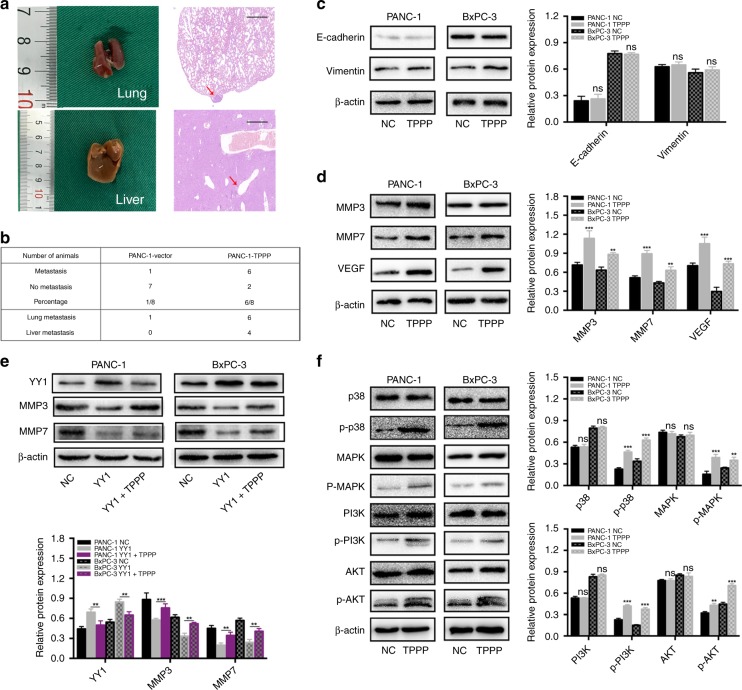
**a** PANC-1 vector and PANC-1-TPPP cells (1.5 × 10^6^ cells/100 μl) were separately injected into the tail vein of each mouse. Four weeks later, lung and liver metastases were evaluated by macroscopic observation and by histomorphology under microscopy. Scale bar = 200 μm. The arrows indicate the metastases. **b** Table listing the incidence of metastases in the nude mice treated with the vector or TPPP. **c** There were no statistically significant differences in the expression of EMT signalling pathway-related proteins between the two groups. **d** Effects of TPPP and its corresponding control group on MMP3, MMP7 and VEGF expression. **e** Effects of YY1, YY1 + TPPP and their corresponding control groups on MMP3 and MMP7 expression. **f** The expression of p38, p-p38, MAPK, p-MAPK, PI3K, p-PI3K, AKT and p-AKT in PANC-1 and BxPC-3 cells after TPPP overexpression

Our observation that the overexpression of TPPP promoted the migration and invasion of pancreatic cancer cells prompted us to investigate its role in vivo. Stable cells were injected into the tail veins of BALB/c-nu nude mice that were killed after 4 weeks of observation. The lung or liver metastasis was observed in six of the eight mice injected with PANC-1-TPPP cells (Fig. 5a). In contrast, only one of the eight mice injected with PANC-1-vector cells exhibited metastasis (Fig. 5b). There was a significant difference between metastasis in the PANC-1-TPPP group and that in the PANC-1-vector group (*p* = 0.044). These results indicate that TPPP promotes the migration and invasion of pancreatic cancer cells.

### TPPP regulates the p38/MAPK and PI3K/AKT signalling pathways

Functional experiments have shown that TPPP overexpression promotes the migration and invasion of pancreatic cancer cells, but this mechanism requires further exploration. Numerous studies have shown that abnormal epithelial–mesenchymal transition (EMT) and expression of members of the matrix metalloproteinases (MMPs) are common mechanisms involved in cancer cell metastasis.^25,26^

As shown in Fig. 5c, E-cadherin and vimentin (markers of epithelial–mesenchymal transition) were not significantly different in the TPPP overexpression group and the control group. Therefore, EMT may not be involved in the TPPP-induced migration and invasion of pancreatic cancer cells. Moreover, compared with their expression in the control group, we found that the expression of MMP3 and MMP7 were increased in TPPP-overexpressing cells. In addition, we also verified the expression of MMPs in the PANC-1-YY1 and BxPC-3-YY1 cell lines. The results showed that overexpression of YY1 decreased the expression of MMP3 and MMP7, while overexpression of TPPP partially restored the inhibitory effect of YY1 on MMPs. These results indicate that MMPs may be involved in YY1 targeting the TPPP-induced migration and invasion of pancreatic cancer cells. Furthermore, VEGF is currently the most potent angiogenesis-inducing factor known to be involved in tumour angiogenesis.^27^ In TPPP-overexpressing cells, we found that the expression of VEGF was increased compared with its expression in control cells. The ability of TPPP to promote angiogenesis in pancreatic cancer cells may be related to VEGF (Fig. 5d, e).

The p38 and PI3K pathways are classical pathways involved in the migration and invasion of cancer, and p38 and PI3K are also the key nodes regulating the expression of MMPs.^28,29^ We detected the total and phosphorylated expression levels of the p38, MAPK, PI3K and AKT proteins by western blot. The phosphorylation of members of the p38/MAPK and PI3K/AKT pathways was significantly increased in PANC-1-TPPP and BxPC-3-TPPP cells compared with their phosphorylation in the corresponding control cells (Fig. 5f). These results suggest that the p38/MAPK and PI3K/AKT pathways may play an important role in the migration and invasion of pancreatic cancer cells induced by TPPP.

## Discussion

Pancreatic cancer is particularly discouraging for many researchers because of its high invasiveness and poor prognosis.^30^ It is especially important to explore new therapeutic targets for PDAC. In this study, we found that TPPP is underexpressed in pancreatic cancer tissues closely related to blood vessel invasion. In addition, TPPP promoted the migration, invasion and angiogenesis of pancreatic cancer cells, and was regulated by the transcription factor YY1.

The transcription factor YY1 is involved in the progression of a variety of tumours, such as prostate cancer, ovarian cancer and colorectal cancer.^31–33^ The overexpression of YY1 in pancreatic cancer can inhibit the invasion and migration of pancreatic cancer.^10^ Using luciferase and ChIP assays, we demonstrated that YY1 binds to the promoter region of TPPP and inhibits the transcription of TPPP in vivo. According to the results of qRT-PCR and WB, TPPP was overexpressed when YY1 was knocked down. These results indicate that TPPP regulates pancreatic cancer migration, invasion and angiogenesis by YY1.

As a member of the tubulin polymerisation-promoting protein family, TPPP can regulate microtubule systems with tubulin/microtubules as its targets.^34^ TPPP is cloned by the human hypothalamic–pituitary–adrenal axis. Studies have shown that the downregulation of TPPP may affect the degree of promoter methylation and tumour development.^19,35^ Through in vitro cell experiments and in vivo animal experiments, we found that TPPP overexpression might promote the migration and invasion of pancreatic cancer. As common genes involved in tumour migration and invasion, MMPs change the tumour microenvironment, activate proteolytic enzyme activity, and degrade the extracellular matrix.^26^ According to our WB results, we found that the expression of MMP3 and MMP7 increased when TPPP was overexpressed. Moreover, we found that YY1 overexpression reduced the expression of MMP3 and MMP7, whereas TPPP partially restored this inhibition. Taken together, MMP3 and MMP7 might be involved in the migration and invasion of pancreatic cancer induced by YY1 targeting TPPP. P38 is expressed in a variety of cell types and regulates a variety of physiological processes. P38 can activate MAPK to promote tumour progression.^36^ As the main downstream effector of PI3K, AKT is often dysregulated in tumour progression.^37^ Drugs targeting PI3K or AKT have been developed for tumour treatment.^38^ After the overexpression of TPPP, we found that the phosphorylation of p38/MAPK and PI3K/AKT were increased. These results indicated that the p38/MAPK and PI3K/AKT signalling pathways might play important roles in the TPPP-induced migration and invasion of pancreatic cancer.

In conclusion, our study revealed the low expression level of TPPP in pancreatic cancer cells. In addition, we also found that the high expression of TPPP promotes the invasion, migration and angiogenesis of pancreatic cancer cells. As a transcription factor, YY1 can directly bind to the promoter region of TPPP and inhibit the expression of TPPP. This evidence suggests that TPPP might be a new target for treating pancreatic cancer.

## Supplementary information

Supplementary File

## Data Availability

All data generated or analysed during this study are included in this published article.
