# Supplementary material for: YY1 targets tubulin polymerisation-promoting protein to inhibit migration, invasion and angiogenesis in pancreatic cancer via p38/MAPK and PI3K/AKT pathways
Source: Br J Cancer. 2019 Oct 21;121(11):912–21. doi: 10.1038/s41416-019-0604-5 (PMC6888832; doi:10.1038/s41416-019-0604-5)

**Legends**

Supporting Information Table S1. qPCR Primers used for detecting miRNAs expression.

Supporting Information Table S2. TPPP expression in tissue microarrays.

Supporting Information Table S3. Clinical information of patients with tissue microarrays.

Supporting Information Table S4. NC3Rs ARRIVE Guidelines Checklist.

**Table S1. qPCR Primers used for detecting miRNAs expression.**

| Genes | Primer sequence(5’-3’) |
| --- | --- |
| TPPP | Forward:5’- ATTCAAAGACAAGAGCAGCGA -3’  Reverse:5’- GCTTTCGTCACCCCTGAGATG -3’ |
| YY1 | Forward:5’-ACGGCTTCGAGGATCAGATTC-3’  Reverse:5’-TGACCAGCGTTTGTTCAATGT -3’ |
| β-actin | Forward:5’-AGAAAATCTGGCACCACACC-3’  Reverse:5’-TAGCACAGCCTGGATAGCAA-3’ |

**Table S2. TPPP expression in tissue microarrays.**

| **Number** | **Type of tissue** | **Cytolymph** | | **Cytomembrane** | | **Nucleus** | |
| --- | --- | --- | --- | --- | --- | --- | --- |
|  |  | **intensity score** | **positive rate score** | **intensity score** | **positive rate score** | **intensity score** | **positive rate score** |
| 1 | PDAC | 0 |  | 0 |  | 0 |  |
|  | Normal | 0.5~1 | 20% |  |  |  |  |
| 2 | PDAC | 0 |  | 0 |  | 0 |  |
|  | Normal | 0.5~1 | 60% |  |  |  |  |
| 3 | PDAC | 0.5~1 | 40% |  |  |  |  |
|  | Normal |  |  |  |  |  |  |
| 4 | PDAC | 0 |  | 0 |  | 0 |  |
|  | Normal | 0.5~2 | 25% |  |  |  |  |
| 5 | PDAC | 0.5~1 | 5% |  |  |  |  |
|  | Normal | 0.5~1 | 40% |  |  |  |  |
| 6 | PDAC | 0.5~1 | 50% |  |  |  |  |
|  | Normal | 0.5~2 | 40% |  |  |  |  |
| 7 | PDAC | 0 |  | 0 |  | 0 |  |
|  | Normal | 1 | 90% |  |  |  |  |
| 8 | PDAC | 0 |  | 0 |  | 0 |  |
|  | Normal | 1 | 85% |  |  |  |  |
| 9 | PDAC | 0 |  | 0 |  | 0 |  |
|  | Normal | 0.5~1 | 70% |  |  |  |  |
| 10 | PDAC | 0.5~1 | 5% |  |  |  |  |
|  | Normal | 1 | 85% |  |  |  |  |
| 11 | PDAC |  |  |  |  |  |  |
|  | Normal | 0.5~1 | 65% |  |  |  |  |
| 12 | PDAC | 0 |  | 0 |  | 0 |  |
|  | Normal | 0.5~1 | 40% |  |  |  |  |
| 13 | PDAC | 0 |  | 0 |  | 0 |  |
|  | Normal | 1 | 85% |  |  |  |  |
| 14 | PDAC | 0 |  | 0 |  | 0 |  |
|  | Normal | 0.5~1 | 60% |  |  |  |  |
| 15 | PDAC | 0 |  | 0 |  | 0 |  |
|  | Normal | 0.5~1 | 70% |  |  |  |  |
| 16 | PDAC | 0 |  | 0 |  | 0 |  |
|  | Normal | 0.5~1 | 30% |  |  |  |  |
| 17 | PDAC | 0 |  | 0 |  | 0 |  |
|  | Normal | 0.5~1 | 20% |  |  |  |  |
| 18 | PDAC | 0 |  | 0 |  | 0 |  |
|  | Normal | 0.5~1 | 50% |  |  |  |  |
| 19 | PDAC | 0 |  | 0 |  | 0 |  |
|  | Normal | 0.5~1 | <5% |  |  |  |  |
| 20 | PDAC | 0 |  | 0 |  | 0 |  |
|  | Normal | 0.5~1 | <5% |  |  |  |  |
| 21 | PDAC | 0 |  | 0 |  | 0 |  |
|  | Normal | 1 | <5% |  |  |  |  |
| 22 | PDAC | 0.5~1 | 25% | 0 |  | 0 |  |
|  | Normal | 0.5~1 | 10% |  |  |  |  |
| 23 | PDAC | 0 |  |  |  |  |  |
|  | Normal | 0.5~1 | 80% |  |  |  |  |
| 24 | PDAC | 0 |  | 0 |  | 0 |  |
|  | Normal | 0.5~1 | 85% |  |  |  |  |
| 25 | PDAC | 0.5~1 | 40% | 0 |  | 0 |  |
|  | Normal | 0.5~1 | 15% |  |  |  |  |
| 26 | PDAC | 0 |  |  |  |  |  |
|  | Normal | 0.5~1 | 60% |  |  |  |  |
| 27 | PDAC | 0 |  | 0 |  | 0 |  |
|  | Normal | 1 | 85% |  |  |  |  |
| 28 | PDAC | 0 |  | 0 |  | 0 |  |
|  | Normal | 1~2 | <5% |  |  |  |  |
| 29 | PDAC | 0 |  | 0 |  | 0 |  |
|  | Normal | 0.5~1 | 60% |  |  |  |  |
| 30 | PDAC | 0 |  | 0 |  | 0 |  |
|  | Normal | 0.5~1 | 30% |  |  |  |  |
| 31 | PDAC | 0 |  | 0 |  | 0 |  |
|  | Normal | 1 | <5% |  |  |  |  |
| 32 | PDAC | 0 |  | 0 |  | 0 |  |
|  | Normal | 0.5~1 | 70% |  |  |  |  |
| 33 | PDAC | 0 |  | 0 |  | 0 |  |
|  | Normal | 0.5~1 | 40% |  |  |  |  |
| 34 | PDAC | 0.5~1 | 5% | 0 |  | 0 |  |
|  | Normal | 2~3 | 5% |  |  |  |  |
| 35 | PDAC | 0.5~1 | 70% |  |  |  |  |
|  | Normal |  |  |  |  |  |  |
| 36 | PDAC | 0 |  |  |  |  |  |
|  | Normal | 0.5~1 | 85% |  |  |  |  |
| 37 | PDAC | 0 |  | 0 |  | 0 |  |
|  | Normal | 0.5~2 | 85% |  |  |  |  |
| 38 | PDAC | 0 |  | 0 |  | 0 |  |
|  | Normal | 0.5~1 | <5% |  |  |  |  |
| 39 | PDAC | 0 |  | 0 |  | 0 |  |
|  | Normal | 0.5~1 | 90% |  |  |  |  |
| 40 | PDAC | 0 |  | 0 |  | 0 |  |
|  | Normal | 0.5~1 | 90% |  |  |  |  |
| 41 | PDAC | 0 |  | 0 |  | 0 |  |
|  | Normal | 0.5~1 | 70% |  |  |  |  |
| 42 | PDAC | 0 |  | 0 |  | 0 |  |
|  | Normal | 0.5~2 | 90% |  |  |  |  |
| 43 | PDAC | 0 |  | 0 |  | 0 |  |
|  | Normal | 1~2 | 90% |  |  |  |  |
| 44 | PDAC | 0 |  | 0 |  | 0 |  |
|  | Normal | 1~3 | 5% |  |  |  |  |
| 45 | PDAC | 0 |  | 0 |  | 0 |  |
|  | Normal | 0.5~1 | 40% |  |  |  |  |
| 46 | PDAC | 0 |  | 0 |  | 0 |  |
|  | Normal | 1 | 95% |  |  |  |  |
| 47 | PDAC | 1 | 80% | 0 |  | 0 |  |
|  | Normal | 1~2 | 10% |  |  |  |  |
| 48 | PDAC | 0 |  | 0 |  | 0 |  |
|  | Normal | 1~3 | 10% |  |  |  |  |
| 49 | PDAC | 0 |  | 0 |  | 0 |  |
|  | Normal | 0.5~1 | 25% |  |  |  |  |
| 50 | PDAC | 0 |  | 0 |  | 0 |  |
|  | Normal | 0.5~1 | 20% |  |  |  |  |
| 51 | PDAC | 0 |  | 0 |  | 0 |  |
|  | Normal | 0.5~1 | 40% |  |  |  |  |
| 52 | PDAC | 0 |  | 0 |  | 0 |  |
|  | Normal | 1~3 | 10% |  |  |  |  |
| 53 | PDAC | 0 |  | 0 |  | 0 |  |
|  | Normal | 0.5~1 | 40% |  |  |  |  |
| 54 | PDAC |  |  |  |  |  |  |
|  | Normal | 0.5~1 | 30% |  |  |  |  |
| 55 | PDAC | 0 |  | 0 |  | 0 |  |
|  | Normal | 0.5~1 | 70% |  |  |  |  |
| 56 | PDAC |  |  |  |  |  |  |
|  | Normal | 0.5~1 | 15% |  |  |  |  |
| 57 | PDAC | 0 |  | 0 |  | 0 |  |
|  | Normal | 0.5~1 | 30% |  |  |  |  |
| 58 | PDAC | 0 |  | 0 |  | 0 |  |
|  | Normal | 1~2 | 10% |  |  |  |  |
| 59 | PDAC | 0.5~1 | 5% |  |  |  |  |
|  | Normal | 0.5~1 | 30% |  |  |  |  |
| 60 | PDAC | 0.5~1 | 20% |  |  |  |  |
|  | Normal | 0.5~1 | 85% |  |  |  |  |
| 61 | PDAC | 0 |  | 0 |  | 0 |  |
|  | Normal | 0.5~2 | 80% |  |  |  |  |
| 62 | PDAC | 0 |  | 0 |  | 0 |  |
|  | Normal | 1~3 | 15% |  |  |  |  |
| 63 | PDAC | 0 |  | 0 |  | 0 |  |
|  | Normal | 0.5~2 | 90% |  |  |  |  |
| 64 | PDAC | 0 |  | 0 |  | 0 |  |
|  | Normal | 0.5~1 | 60% |  |  |  |  |
| 65 | PDAC | 1~2 | 90% |  |  |  |  |
|  | Normal | 1~3 | 50% |  |  |  |  |
| 66 | PDAC | 0 |  | 0 |  | 0 |  |
| 67 | PDAC | 1 | 80% |  |  |  |  |
| 68 | PDAC | 0 |  | 0 |  | 0 |  |
| 69 | PDAC | 0 |  | 0 |  | 0 |  |
| 70 | PDAC | 0 |  | 0 |  | 0 |  |
| 71 | PDAC | 0 |  | 0 |  | 0 |  |

**Table S3. Clinical information of patients with tissue microarrays.**

| **Number** | **Gender** | **Age** | **Histological grade** | **Diameter (cm)** | **Location** | **Blood vessel invasion** | **T** | **N** | **M** | **TNM stage** |
| --- | --- | --- | --- | --- | --- | --- | --- | --- | --- | --- |
| 1 | Male | 84 | II-III | 2×1.5×1.5 | Head | Absent | T1 | N0 | M0 | IA |
| 2 | Female | 78 | II | 3.5×2.5×2.5 | Head | Absent | T2 | N0 | M0 | IB |
| 3 | Female | 77 | I | 4×4×3 | Head | Absent | T2 | N0 | M0 | IB |
| 4 | Female | 77 | II-III | 3.5×3×3 | Head | Absent | T2 | N0 | M0 | IB |
| 5 | Male | 72 | II-III | 4×3.5×3.5 | Body, Tail | Absent | T2 | N0 | M0 | IB |
| 6 | Female | 72 | I-II | 4×3×3 | Body, Tail | Absent | T2 | N1 | M1 | IV |
| 7 | Male | 71 | II-III | 4×3×3 | Head | Absent | T2 | N0 | M0 | IB |
| 8 | Male | 71 | II | 4×3.5×3 | Head | Present | T2 | N1 | M0 | IIB |
| 9 | Male | 71 | II | 4×3.5×3 | Head | Present | T2 | N1 | M0 | IIB |
| 10 | Female | 70 | II | 2×2×2 | Head | Absent | T1 | N0 | M0 | IA |
| 11 | Female | 70 | II | 6×5×3 | Head | Absent | T3 | N0 | M0 | IIA |
| 12 | Male | 69 | II-III | 4×4×3 | Body, Tail | Present | T2 | N0 | M0 | IB |
| 13 | Male | 69 | II | 4×4×3.5 | Head | Present | T2 | N1 | M0 | IIB |
| 14 | Male | 65 | II | 2×2×2 | Head | Absent | T1 | N0 | M0 | IA |
| 15 | Male | 65 | II | 4×2×1 | Head | Present | T2 | N0 | M0 | IB |
| 16 | Male | 65 | III | 4×4×3.5 | Body, Tail | Present | T2 | N1 | M0 | IIB |
| 17 | Male | 65 | III | 2.5×2×1.5 | Head | Absent | T2 | N1 | M0 | IIB |
| 18 | Male | 64 | II | 4×3×2 | Body, Tail | Present | T2 | N0 | M0 | IB |
| 19 | Male | 64 | II | 3.5×2×2 | Body, Tail | Absent | T2 | N0 | M0 | IB |
| 20 | Male | 64 | II | 3×3×2 | Body, Tail | Absent | T2 | N0 | M0 | IB |
| 21 | Male | 64 | II | 1×1×0.5 | Head | Present | T1 | N1 | M0 | IIB |
| 22 | Male | 64 | II | 4×4×3 | Head | Absent | T2 | N1 | M0 | IIB |
| 23 | Male | 64 | I-II | 3.5×3×1 | Head | Present | T2 | N0 | M0 | IB |
| 24 | Male | 63 | III | 4×3×2 | Body, Tail | Present | T2 | N0 | M0 | IB |
| 25 | Female | 63 | II | 3×3×2.5 | Head | Absent | T2 | N0 | M0 | IB |
| 26 | Female | 63 | II | 2×1.5×1 | Head | Absent | T1 | N0 | M0 | IA |
| 27 | Female | 63 | II | 5×4×3 | Head | Present | T3 | N0 | M0 | IIA |
| 28 | Male | 62 | I-III | 3.5×3×3 | Body | Present | T2 | N0 | M0 | IB |
| 29 | Male | 62 | I-II | 6×5×4 | Head | Present | T3 | N1 | M0 | IIB |
| 30 | Male | 62 | II | 4×4×3 | Body, Tail | Absent | T2 | N0 | M1 | IV |
| 31 | Male | 62 | II-III | 2.5×2×1 | Head | Present | T2 | N0 | M0 | IB |
| 32 | Female | 62 | II | 2.5×2×2 | Tail | Present | T2 | N0 | M0 | IB |
| 33 | Female | 62 | II | 3×2×1 | Head | Absent | T2 | N0 | M0 | IB |
| 34 | Female | 62 | II-III | 4×3×3 | Head | Absent | T2 | N0 | M0 | IB |
| 35 | Male | 61 | II | 3.5×3×2.5 | Head | Present | T2 | N0 | M0 | IB |
| 36 | Male | 61 | II-III | 3.5×2×2 | Head | Present | T2 | N0 | M0 | IB |
| 37 | Male | 61 | III | 4×4×4 | Head | Absent | T2 | N1 | M0 | IIB |
| 38 | Female | 61 | III | 2×1×1 | Head | Absent | T1 | N0 | M0 | IA |
| 39 | Male | 60 | II-III | 4×3×2.5 | Head | Present | T2 | N0 | M0 | IB |
| 40 | Female | 60 | II-III | 2.5×2×1.5 | Head | Absent | T2 | N1 | M0 | IIB |
| 41 | Female | 60 | III | 3×3×2.5 | Body, Tail | Absent | T2 | N0 | M1 | IV |
| 42 | Female | 60 | II | 3.5×3×3 | Head | Present | T2 | N1 | M1 | IV |
| 43 | Male | 58 | III | 3×2×2 | Head | Present | T2 | N0 | M0 | IB |
| 44 | Male | 57 | II | 3.5×2.5×2 | Body, Tail | Absent | T2 | N0 | M0 | IB |
| 45 | Female | 57 | II | 2.5×1×1 | Body, Tail | Present | T2 | N0 | M0 | IB |
| 46 | Female | 57 | II | 3×2×1.5 | Head | Present | T2 | N1 | M0 | IIB |
| 47 | Male | 55 | II-III | 4×3.5×2.5 | Head | Absent | T2 | N0 | M0 | IB |
| 48 | Male | 55 | II | 2×2×1 | Head | Absent | T1 | N0 | M0 | IA |
| 49 | Male | 55 | II-III | 4×3×2 | Head | Absent | T2 | N1 | M1 | IV |
| 50 | Female | 55 | II | 3×2.5×2 | Head | Present | T2 | N0 | M0 | IB |
| 51 | Female | 55 | II | 3×3×3 | Head | Present | T2 | N1 | M0 | IIB |
| 52 | Male | 54 | II | 3×2.5×2 | Body, Tail | Absent | T2 | N0 | M0 | IB |
| 53 | Male | 53 | II-III | 5×5×1 | Head | Absent | T3 | N1 | M1 | IV |
| 54 | Male | 52 | II | 3×2×1 | Head | Present | T2 | N0 | M0 | IB |
| 55 | Male | 52 | II | 4×4×3 | Head | Absent | T2 | N0 | M0 | IB |
| 56 | Male | 52 | II-III | 9.5×4.5×2.5 | Body, Tail | Present | T3 | N0 | M0 | IIA |
| 57 | Male | 52 | II | 4×2×2 | Body | Absent | T2 | N1 | M0 | IIB |
| 58 | Female | 52 | II | 3.5×2.5×2 | Body, Tail | Present | T2 | N0 | M0 | IB |
| 59 | Female | 52 | II | 2×1.5 | Head | Present | T1 | N1 | M1 | IV |
| 60 | Male | 51 | II | 2×2×1.2 | Head | Absent | T1 | N0 | M0 | IA |
| 61 | Male | 51 | II | 4×4×3 | Head | Present | T2 | N0 | M0 | IB |
| 62 | Male | 49 | II-III | 3×2.5×2 | Head | Absent | T2 | N0 | M0 | IB |
| 63 | Male | 48 | II | 3×3×2 | Head | Present | T2 | N0 | M0 | IB |
| 64 | Male | 46 | I-II | 3×2×2 | Head | Absent | T2 | N0 | M0 | IB |
| 65 | Female | 46 | II | 5×2.5×2.5 | Body, Tail | Absent | T3 | N0 | M0 | IIA |
| 66 | Male | 44 | I-II | 5×4×3 | Head | Absent | T3 | N0 | M0 | IIA |
| 67 | Female | 44 | III | 3.5×3×2.8 | Head | Absent | T2 | N0 | M0 | IB |
| 68 | Female | 42 | II | 5.5×3.5×2 | Body, Tail | Absent | T3 | N1 | M0 | IIB |
| 69 | Male | 41 | II-III | 4×3×2 | Head | Absent | T2 | N1 | M0 | IIB |
| 70 | Male | 72 | II | 4×3×3 | Body | Absent | T2 | N1 | M0 | IIB |
| 71 | Female | 79 | I-II | 4×3×2 | Head | Absent | T2 | N1 | M0 | IIB |

**Table S4. NC3Rs ARRIVE Guidelines Checklist.**


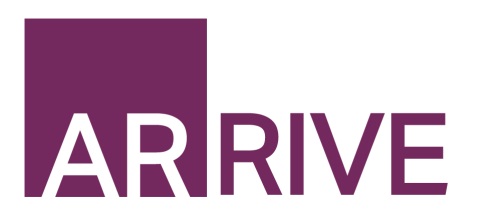


The ARRIVE Guidelines Checklist

Animal Research: Reporting In Vivo Experiments

Carol Kilkenny^1^, William J Browne^2^, Innes C Cuthill^3^, Michael Emerson^4^ and Douglas G Altman^5^

*^1^The National Centre for the Replacement, Refinement and Reduction of Animals in Research, London, UK, ^2^School of Veterinary Science, University of Bristol, Bristol, UK, ^3^School of Biological Sciences, University of Bristol, Bristol, UK, ^4^National Heart and Lung Institute, Imperial College London, UK, ^5^Centre for Statistics in Medicine, University of Oxford, Oxford, UK.*

|  | | ITEM | RECOMMENDATION | Section/ Paragraph |
| --- | --- | --- | --- | --- |
| 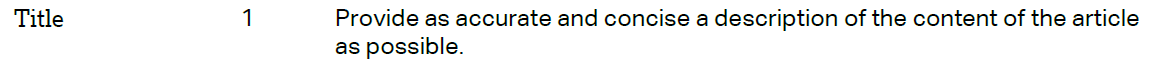 | | | Title, Line 1-2 |  |
| 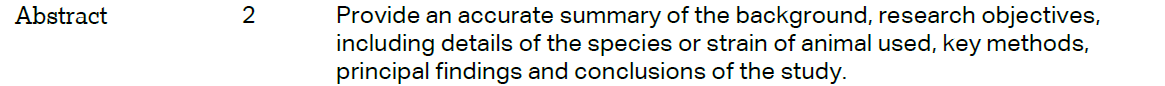 | | | Abstract, paragraph 1-3, Line 16-27 |  |
| INTRODUCTION | | |  |  |
| 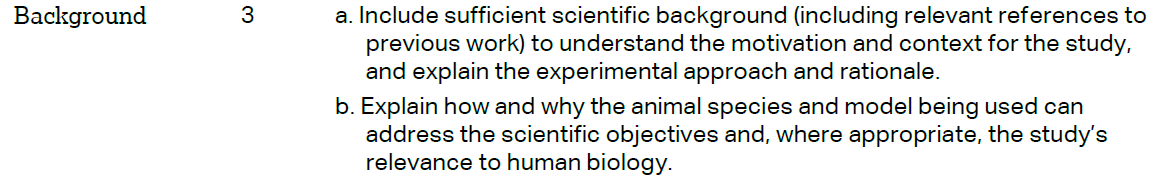 | | | Background, paragraph 1-3, Line 36-54 |  |
| 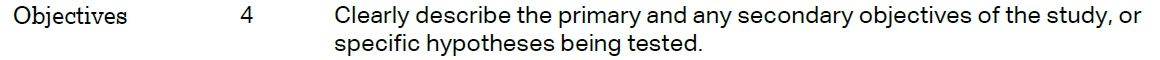 | | | Background, paragraph 4, Line 55-57 |  |
| METHODS | | |  |  |
| 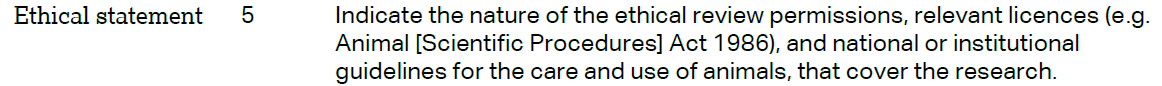 | | | Methods, paragraph 13, Line 167-168 |  |
| 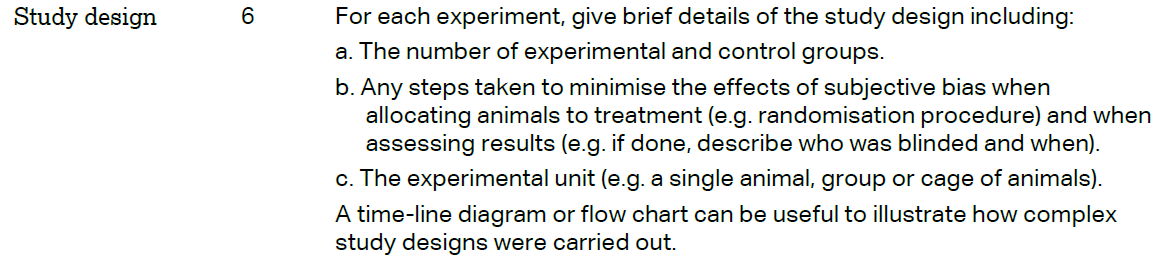 | | | Methods, paragraph 13, Line 169-173 |  |
| 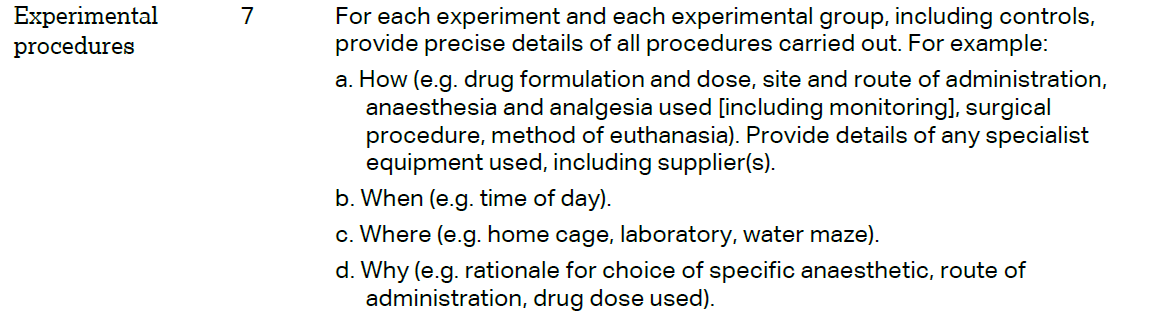 | | | Methods, paragraph 13, Line 169-175 |  |
| 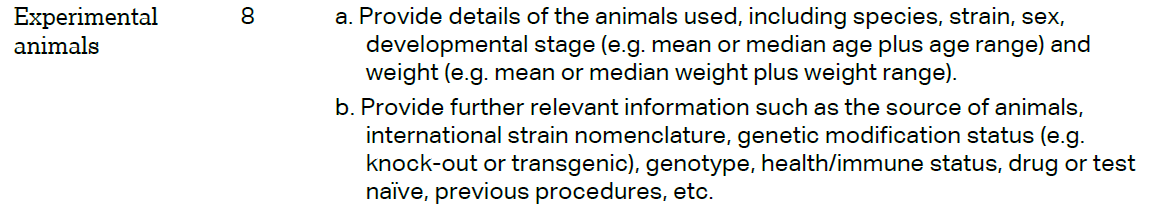 | | | Methods, paragraph 13, Line 166-167 |  |

The ARRIVE guidelines. Originally published in *PLoS Biology*, June 2010^1^

| 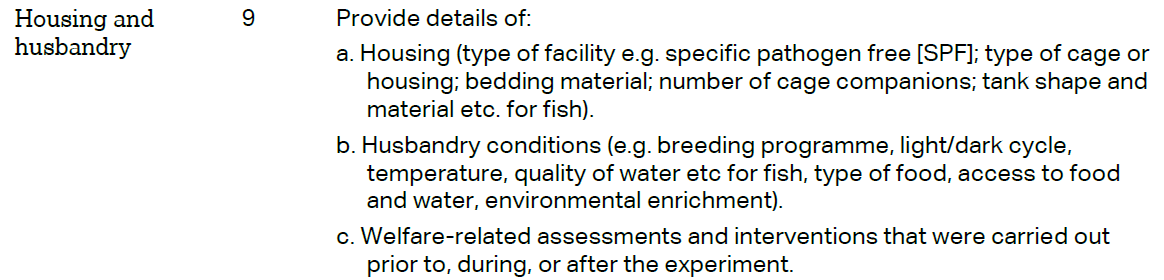 | Methods, paragraph 13, Line 168-169 | |
| --- | --- | --- |
| 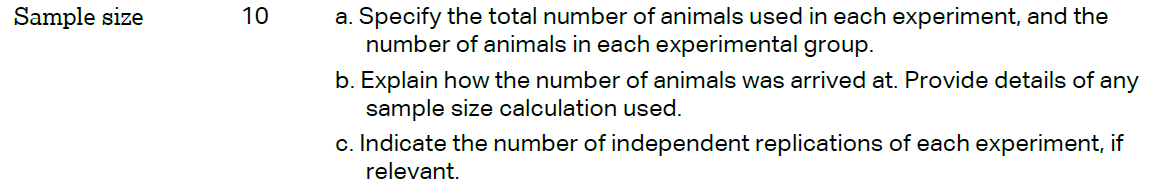 | Methods, paragraph 13, Line 169-170 | |
| 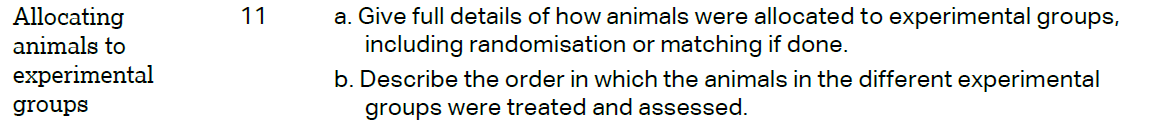 | Methods, paragraph 13, Line 169-170 | |
| 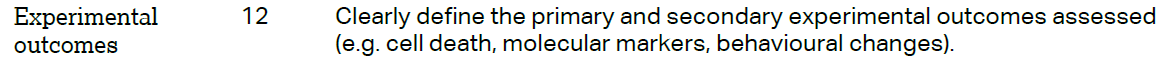 | Methods, paragraph 13, Line 173-175 | |
| 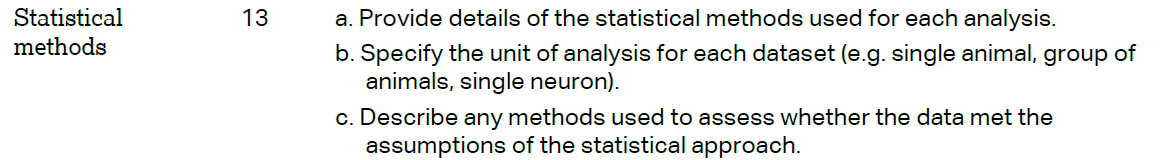 | Methods, paragraph 14, Line 177-181 | |
| RESULTS |  | |
| 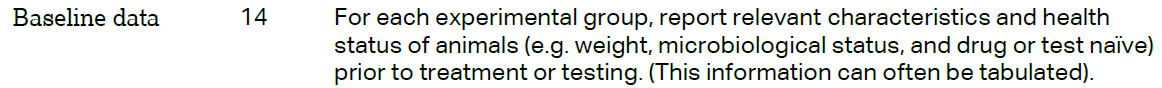 | Results, paragraph 5, Line 244-247 | |
| 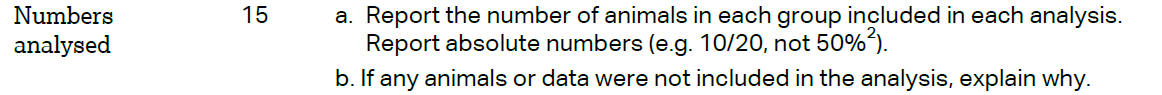 | Results, paragraph 5, Line 246-248 | |
| 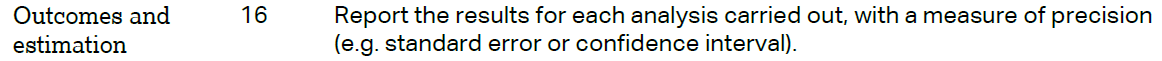 | Results, paragraph 5, Line 247-251 | |
| 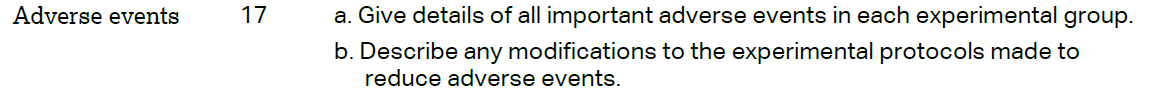 | Results, paragraph 5, Line 246-248 | |
| DISCUSSION |  | |
| 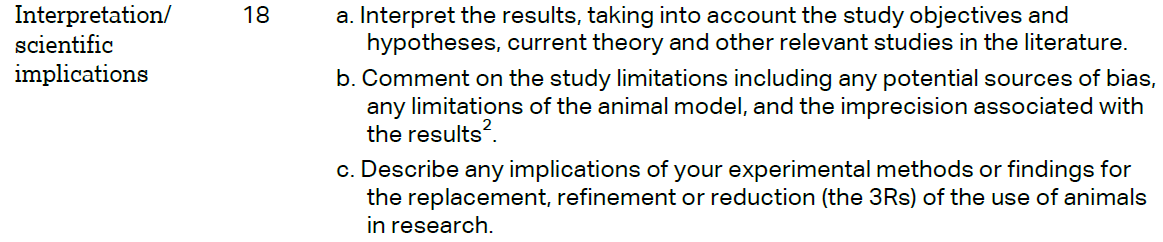 | Discussion, paragraph 1-3, Line 280-297 | |
| 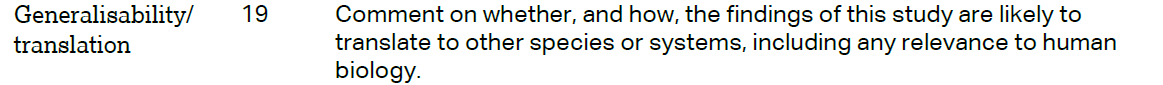 | Discussion, paragraph 3, Line 295-297 | |
| 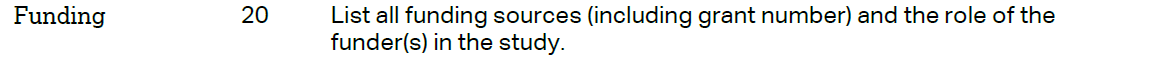 | | Funding, Line 327-331 |


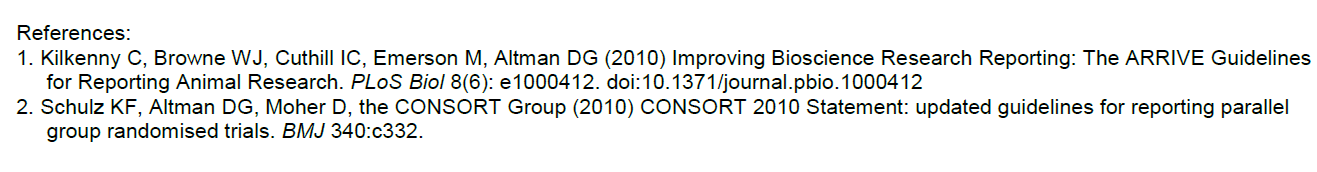

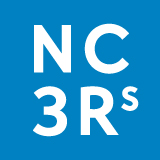

Supplement: Supplementary file 1 — Supplementary File [file 41416_2019_604_MOESM1_ESM.docx]
